# COVID-19 and the future of work and organisational psychology

**DOI:** 10.4102/sajip.v47i0.1854

**Published:** 2021-05-14

**Authors:** Amalia Pérez-Nebra, Chrysavgi Sklaveniti, Gazi Islam, Ivana Petrović, Jennifer Pickett, Makfire Alija, P. Matthijs Bal, Milena Tekeste, Milica Vukelić, Sandiso Bazana, Zoe Sanderson

**Affiliations:** 1 Department of Administration, University of Brasília, Brasilia, Brazil; 2 Institute of Organizational Psychology, School of Management, University of St. Gallen, St. Gallen, Switzerland; 3 Department of People, Organizations and Society, Grenoble Ecole de Management, Grenoble, France; 4 Institute for Research in Management and Economics (IREGE), Savoie Mont Blanc University, Geneva, Switzerland; 5 Department of Psychology, Faculty of Philosophy, University of Belgrade, Belgrade, Serbia; 6 Department of Psychology, Faculty of Psychology and Educational Sciences, Vrije Universiteit Brussel, Brussels, Belgium; 7 Bruegel Think Tank, Brussels, Belgium; 8 Lincoln International Business School, Brayford Wharf, University of Lincoln, Lincoln, United Kingdom; 9 School of Business and Management, Faculty of Organisation Studies, Royal Holloway University of London, London, United Kingdom; 10 Department of Psychology, Faculty of Humanities, Rhodes University, Grahamstown, South Africa; 11 School of Management, Social Sciences and Law, University of Bristol, Bristol, United Kingdom

**Keywords:** COVID-19, corona, work and organisational psychology, theory, neglected perspectives

## Abstract

**Orientation:**

The coronavirus disease 2019 (COVID-19) pandemic has caused a ‘coronafication’ of research and academia, including the instrumentalisation of academic research towards the demands of society and governments. Whilst an enormous number of special issues and articles are devoted on the topic, there are few fundamental reflections on how the current pandemic will affect science and work and organisational psychology in the long run.

**Research purpose:**

The current overview, written by a group of members of the Future of Work and Organisational Psychology (FOWOP) Movement, focuses on the central issues relating to work and organisational psychology that have emerged as a result of the COVID-19 crisis.

**Motivation for the study:**

The study discusses the inability of dominant theories in work and organisational psychology to understand contemporary problems and the need to advance the theoretical realm of work psychology. We also discuss the need for pluralism in methodologies to understand the post-COVID-19 workplace, the urgency of attending to neglected voices and populations during the COVID-19 crisis and teaching during COVID-19.

**Research approach/design and method:**

This article uses conceptual argumentation.

**Main findings:**

The COVID-19 crisis forces work psychology to address at least its theorising, methods, unheard voices and teaching in the COVID-19 crisis.

**Practical/managerial implications:**

On the basis of this article, researchers and practitioners may be better aware of the neglected perspectives in the current pandemic.

**Contribution/value-add:**

This article adds to the understanding of the future directions for a sustainable Work and Organisational Psychology as an applied scientific discipline during and beyond the COVID-19 crisis.

## Introduction

Academic research is currently undergoing what could be called a ‘coronafication’. The focus of research has dramatically shifted to study the effects of the coronavirus disease 2019 (COVID-19) on people globally. Hundreds of articles are currently published in which every existing theory or concept is related to COVID-19. Numerous explanations are given about how the current pandemic is affecting people, workplaces, humanity and the planet (Bapuji, Patel, Ertug, & Allen, 2020; Delanty, 2020). This article, whilst part of this trend, distinguishes itself from the tide of research on COVID-19 by focusing on the implications of our current crisis on future perspectives in work and organisational psychology (henceforth work psychology). The article sets out to engage with what is *not* yet there in understanding how COVID-19 currently unfolds in global society and in particular the emerging world of work.

Whilst pandemics are nothing new and have occurred throughout history, we can observe similarities with previous epidemics, such as those narrated by authors like Daniel Defoe (in his narrative of the plague of 1665 in England) and Albert Camus (in his fictional narrative of a plague in an Algerian city, written in the 1940s). Yet, it can be stated that global society (and especially Western countries) had unlearnt (i.e. forgotten to learn from history and previous epidemics) how to cope with pandemics, as global health pandemics such as the Spanish Flu occurred over a 100 years ago. The world has not faced similar global health crisis since then (e.g. the human immunodeficiency virus/acquired immune deficiency syndrome [HIV/AIDS] pandemic was not as contagious as COVID-19 is).

A global crisis such as the COVID-19 pandemic also reminds us of other (non-epidemic) crises, such as the global economic crisis of 2008–2009, the cultural collapse of the United States of America after 9/11 and others across the world. These crises tend to be intertwined with each other and often provoke each other. This is also currently the case with COVID-19 leading to a global economic crisis, which then leads to a deeper health crisis. Such crises, whether economic or health crises, call for a *radical transformation* of society and its practices and crisis theorists have often argued how crises may provide spaces for radical change (Van de Ven & Poole, 1995).

In the workplace, and especially in formal work, we have seen such radical measures taken in response to the COVID-19 crisis. For instance, whilst many countries and organisations have been reluctant to allow people to work from home, during the present crisis, they have rapidly adjusted to the necessity of homeworking. People needed to stay at home and balance their ongoing work obligations with taking care of family members and other duties. Consequently, homeworking has suddenly become a norm in some organisations and some jobs and people have to rapidly adjust to this new situation. However, Defoe (1722/2001) already wrote about the poor people, maids and servants who were most vulnerable and exposed to the plague in 1665, as they did not have the choice to stay at home and wait until the plague was over. Instead, they were forced, because of economic reasons, to expose themselves to the possibility of contracting the plague. Seemingly, not much has changed in these 400 years (e.g. Bargain & Aminjonov, 2020).

Discussions about homeworking, prevalent in recent work psychology literature (e.g. Chong, Huang, & Chang, 2020), indicate the elitist nature of academic thinking around people at work. Such elitist and exclusive perspectives are deeply engrained in the work psychology literature. Many responses to COVID-19 published in the field have aligned with such elitist work perspectives, including the focus on working from home, virtual teamwork and virtual leadership and management. Such work takes for granted the elitist assumption that for many across the world, homeworking and virtual working are impossible and only pertain to a privileged minority.

Moreover, the literature has also focused mainly on formal work in the formal employment sector. In this way, lessons from the informal sector are ignored, which is the part of the economy that is neither taxed nor monitored by the government (e.g. Kim, 2019; Sudarshan & Sinha, 2011). Therefore, it is important to raise more broad-ranging issues pertaining to the impact of the crisis and how the future of work psychology can be shaped in a more sustainable way. This includes taking into account the needs of all workers globally, and not just those few in the Global North who have the privilege of having a job and space at home, which allows them to continue their work activities.

In this article, we will discuss the implications of the COVID-19 crisis for work psychology, with special interest in what has been excluded from previous discussions on the impact of the crisis. We will do so by discussing three main areas within the field that need to be better understood in the COVID-19 workplace context. We discuss implications for (1) theorising in work psychology, (2) methodology, (3) the role of unheard voices and (4) teaching in work psychology. The article will finish with a discussion of the implications for the future of work psychology, which is heavily based on the manifesto for the Future of Work and Organisational Psychology (FOWOP) (Bal et al., 2019).

## Theorising in times of COVID-19

Researchers across the world have tried to understand the impact of the current crisis on people and workplaces. They have largely done so through applying existing concepts and theories to the COVID-19 crisis. However, if the current crisis teaches humanity one lesson, it is that existing concepts cease to be able to explain our current predicament against the backdrop of a globalised world of increasing inequalities, dominated by neoliberal capitalism, and disintegrating democracies (Delanty, 2020; Žižek, 2020). It is no longer sufficient to rely on existing theory and concepts about work and its place in society as much of this theorising is woefully outdated and irrelevant when it comes to COVID-19. Triggered by the COVID-19 crisis, three critiques of contemporary theorising in work psychology are offered: work–life balance, the meaning of work and life and the dominance of the individual as a unit of analysis. More context and time-relevant responses by work psychology are suggested in each case.

### Critique 1: Work–life balance

One of the principal features of theory in our field is that it encourages empirical attempts to support theories. Hence, the focus is on theoretical validation rather than Popperian falsification. This focus puts us in a situation *not* to see. We thus ignore what is *not* captured by theories and concepts in our field. For instance, work–life balance theory postulates that people maintain certain boundaries between the world of work and their other life domains, such as private and family life. However, in our current pandemic, theory fails to capture the possibility of *no* boundary at all. Consequently, the entire disappearance of work–life balance may go unnoticed, in cases where the two are indefinitely intertwined.

Still, the principal research direction of our field in times of the pandemic has been the study of work–life balance as it has been disrupted by the crisis. This direction, although significant for the insight it offers about those concerned, is only one voice of the workplace during the pandemic. Whilst it appears as the dominant one, on the one hand, this view of work does not represent a large proportion of the global work population. On the other hand, it is not the only valid voice. Many voices are not heard or considered as relevant.

For some individuals, home has always been the place where work is conducted. The impossibility for them of separating personal life from work is evident. For them, a concept such as work–life balance is absent or irrelevant. For instance, this applies to those who are unable to work from home (which involves many people in the informal sector; Sudarshan & Sinha, 2011), and those who engage in work that has to take place onsite or in direct contact with other people. For them, it no longer suffices to keep one’s private life separate from work, as the two become inherently integrated through the disclosure of personal health information (e.g. an employer may request a negative COVID-19 test in order to work). Moreover, one may need to restrict one’s movements in or outside one’s job to protect oneself and one’s colleagues or family against exposure to the virus. Is the fact that scholars presume that a boundary *should* exist between the work domain and one’s private life (which is a historically recent invention anyway) not indicative of the destructive effects of work in contemporary capitalism on well-being and non-work life?

The fact that people need to (mentally and physically) disengage from work to enter their private lives, indicated by the concept of work–life balance, may also be pointing towards the psychological violence of organisational life that is exercised on the modern individual in contemporary capitalism. Adding to such a problematisation, the focus on the primary discourse of work–life balance and the assumption that a boundary should exist between the work domain and one’s private life gives a negative undertone to the notion of work in contemporary capitalism, operating at the expense of well-being and non-work life.

All of these are connected to long-held work stereotypes that have been challenged during the pandemic, for instance, the compliance to one ideal work model (the traditional full-time mode), one ideal career type, typically involving the gradual advancement in the hierarchical ladder. The field of work psychology is primarily focused on the matter of work–life balance as a major concern for those workers performing work from home (as opposed to the traditional setting of the organisation). In contrast, the possibility of flexible home office had been almost always denied to those workers whose job may be carried out remotely and whose life needs would benefit from it. Notwithstanding the strain that is involved in managing work and parenthood, the experience of the COVID-19 pandemic urges us, work psychologists, to change the tune in working parenthood and move towards a holistic understanding of the diversity of roles and expectations in modern families.

Work–life balance as an issue focuses academic attention on those people who *can* work from home. However, for those who are unable to work from home, balancing work with one’s life seems to be an increasing absurdity in the light of one’s need to survive the crisis. In fact, the crisis may expose people to much more fundamental questions regarding the meaning of life (Blustein & Guarino, 2020) than the rather ‘mundane’ theorising around work–life balance is able to address. It is not surprising to observe a rise of interest in existentialist philosophers and writers, such as Camus, Arendt and Frankl (Blustein & Guarino, 2020). In essence, these authors dealt with the question of the meaning of life in uncertain and volatile times.

Questions such as ‘what to do with my life’ do not merely imply a philosophical depth as to the questions people ask themselves these days. They also have important psychological implications, which are still largely overlooked in the literature. Hence, instead of a focus on how people can maintain their work–life balance, it is time for work psychologists to focus more deeply and ask more relevant questions with respect to the meaning of work in life.

Moreover, this also includes a rethinking of the meaning of the term ‘work’ itself. The concept of work–life balance assumes a separation of paid work and life outside paid work, as if unpaid and household work is unimportant to the work psychologist. Feminist scholars (e.g. Sanchez & Kane, 1996; Sperling, Ferree, & Risman, 2001) have pointed to this artificial gendered distinction for a long time. Work psychologists will have to engage sooner rather than later in this critique, pointing to a blind spot in our current thinking.

From the above discussion it can be concluded that work–life balance theory fails to explain contemporary dynamics between what can be considered the work domain and the private domain. For more than 50% of the global population, COVID-19 may be less of an immediate concern than the everyday occupation of surviving and having enough money to pay for food, bills and accommodation. Such concerns may be exacerbated by the economic impacts of the crisis, independently of the direct effects of disease or illness.

South Africa has a poverty rate of 24% (OECD, 2020). This raises the question of how relevant the dominant, elitist theorising on work–life balance is. In Brazil, which is globally the sixth largest country in terms of population, close to 55 million people earn less than €80 a month and thus live below the poverty line (IBGE, 2018). The coronavirus disease 2019 is likely to extend this number and cause many people worldwide to struggle even more with surviving and getting enough to eat. Discussions about work–life balance in times of COVID-19 may border on the absurd, especially taking into account the unequal distribution of suffering as a result of the crisis across the world. The projection of another rise in extreme poverty resulting from the COVID-19 pandemic is most disturbing (World Bank, 2020).

In summary, whilst there already existed a need to revise the existing theory in work psychology to include discussions of global capitalism and its effects on work psychology (see, e.g., Bal & Dóci, 2018), the current crisis has re-emphasised the urgency to do so. Moreover, most of the ruling theories in work psychology are by nature exclusively Western, educated, industrialised, rich and democratic (WEIRD) orientated, focusing exclusively on concerns of the Global North. They have a high likelihood of failing to explain human behaviour in the workplace in a globalised world (Henrich, Heine, & Norenzayan, 2010; Muthukrishna et al., 2020).

The current crisis offers work psychologists the opportunity to radically rethink our theorising, use and definition of concepts. One criterion for the future of the field could involve considering the global reach of concepts when considering their theoretical validity. For instance, if a work psychologist is interested in the balance employees experience between office life and non-office life, the scope of such an interest could be made explicit. This would lead to an appropriate assessment of the validity of the claims within a given scope. It may be of great interest to study work–life balance during the current COVID-19 crisis in more detail. In addition, it is also crucial to think about what the most pressing issues are that work psychologists *should* study and what our responsibilities as human beings and work psychologists actually are (e.g. Bal et al., 2019).

### Critique 2: The meaning of work and life

In response to the concerns discussed above, one could raise the question of how individuals (worldwide) experience the role of work in their lives. For instance, building on existentialist philosophy, work psychologists can explore the meanings people worldwide draw from work. An increasing part of the working population is merely trying to survive the crisis, in terms that do not acknowledge their existential condition as is done in mainstream work psychology.

Similarly, it is widely known that distancing from each other is the best way to prevent the spreading of the virus. However, the right to such safety conditions remains elusive for many workers worldwide. They are forced to conduct their work in small spaces or on the streets where distancing is not possible. In this way, they are exposing themselves to a greater risk of COVID-19. A need to radically change how we *theorise* in work psychology is eminent in these times of the COVID-19 crisis. Confronted with the predicament of a global pandemic, the potential for the global capitalist system to be disrupted is unprecedented. Where climate change activists have met great resistance in their efforts to call for a sharp reduction in carbon emissions, the pandemic has put an immediate hold on global travel, leading to temporary drops in pollution levels that give a glimmer of hope for a sustainable transformation. In such spirit, work psychology also needs to more radically change how it theorises in order to prioritise humanistic and planetary priorities, rather than the needs of capital in maintaining a global hegemonic order (Bal et al., 2019).

Consequently, we would acknowledge that work psychology should focus on the profound changes people are experiencing in the meaning of work as a result of the current crisis. It has been argued that the crisis has spurred an existentialist experience and questions of loss and fear (Blustein & Guarino, 2020). People are confronted with questions that may have been new to them before the crisis, such as how to survive the coming year, how one’s children will survive if one dies and how to remain healthy and not infected whilst being at work. For others across the world, the crisis may be ‘just another crisis’, such as Serbians who have experienced a long-lasting socio-economic crisis since the 1990s, including the war in former Yugoslavia (e.g. Petrović, Vukelić, & Čizmić, 2017). All of these facts have been largely overlooked so far in work psychology’s responses to where the world is at present.

The crisis also raises even more fundamental questions about the meaning of life and how people can live their lives in a meaningful way. The crisis has spurred a rethinking of what is ‘truly’ important in life. For instance, regarding the concept of ‘bullshit jobs’ (Graeber, 2018), many people may ask themselves in these times of crisis whether their jobs have ‘real’ meaning. However, for others, the meaning is in having a job at all in order to support oneself and one’s family.

### Critique 3: The dominance of the individual as unit of analysis

It has not been surprising that global protest movements, such as the Black Lives Matter (BLM) Movement, have found new energy in the crisis. The crisis has triggered and opened up societal fault lines, such as social injustices, against the backdrop of questioning what is truly important in life. To fight injustices is central to the BLM Movement. However, social justice remains a concept that is undervalued in work psychology, which tends to emphasise more individualistic notions of justice and well-being, rather than collective forms of solidarity.

The current situation is therefore an opportunity for work psychology to revise its main theories, making them less individualistic and more focused on meaning and relationships. ‘Traditional’ work psychology tends to study individuals (or in extension, individuals in teams through a process of theoretical and/or methodological aggregation), whereby individual interest is prioritised in line with the notion of individualism as cornerstone of contemporary society (e.g. Greene, 2008; Markus & Schwartz, 2010). Newly emerging forms of theorising in work psychology could more explicitly focus on both individual and collective levels as units of theorising and analysis. At the same time, researchers may take into account the socially embedded dimensions through which individual and collective behaviour can be properly understood. For instance, dominant perspectives on well-being, originating from North America and Western Europe, tend to emphasise the individualistic bases of well-being (Cederström & Spicer, 2015), built on a variety of personal dimensions (e.g. personal health, optimal working conditions and resilience). However, we do not know enough about how well-being relates to social harmony or collective solidarity. Along similar lines, one cannot merely assume individualistic, hedonic well-being of the modern citizen or employee without taking into account that which unfolds societally (Markus & Schwartz, 2010; Schwartz & Sortheix, 2018; Sortheix & Schwartz, 2017).

Well-being has different referents, including a private (hedonic or eudaimonic) and a public (family, group, hometown, etc.) one. Accordingly, individual well-being cannot be seen separately from social well-being in times of COVID-19, where individual, privileged well-being may occur at the expense of the well-being of others. If the COVID-19 pandemic has taught us anything, it is that we are intrinsically linked to others in aspects as basic as the air we breathe and that conceptions of the autonomous individual are deeply flawed.

In the face of the pandemic the concept of individual responsibility is intrinsically linked to the concept of social solidarity. The guidelines to wear masks, keep physical distance and regularly disinfect our hands are based on the assumptions that we take care not to infect the ones we get in contact with. In such a way, the notion of the individual develops together with others. Togetherness, however, has been surprisingly (maybe not) silenced in work psychology theories. It is now time to consider more relational and collective approaches to theorising and conceptualising human behaviour in the workplace.

A systems approach (such as Bronfenbrenner, 1979; see also Islam, 2020), might offer the possibility to theorise individual behaviour by embedding it in the relational and structural contexts, which shape its meaning and form. To acknowledge the social embeddedness of human behaviour whilst also retaining the lived experience of agents in their everyday concerns, systems approaches could also be integrated with existentialist approaches, with their focus on work and life meanings. These theoretical possibilities may help explain and understand some of the psychologically relevant aspects of the pandemic, including the sharp rise in mental health issues, such as mourning, loss, suicide, isolation, vulnerability, job loss, job insecurity, hunger and fear of infection.

## Methodology in times of COVID-19

Our preceding discussion about the struggles prevailing theorising faces to capture the depth and dynamics of the current COVID-19 crisis naturally leads to problematising methodological approaches apt to include the diversity of discourses around the pandemic. Research in work psychology remains dominated by the positivistic research paradigms, focusing on quantitative research that seeks relationships between variables measured using survey instruments. In critique of the dominance of positivistic research paradigms, we turn to inductive and abductive methodological orientations for exploring experiences and dynamics of the current crisis in inclusive ways that focus on multiplicity rather than dominance of certain discourses, like positivism (both theoretically and empirically observed).

Inductive and abductive research might offer alternative approaches, whereby experiences and dynamics of the current crisis do not have to be fitted in the strict demarcations of existing theories. For instance, whereas job insecurity may result from a wide range of different circumstances, the current pandemic displays a greater volatility, which can be compared with the potential effects of climate change on our lives in the future. The global lockdown and the continued need to reinforce lockdowns worldwide in response to second and third waves of the COVID-19 virus show that perceptions of job insecurity prior to the pandemic do not equate to the insecurity caused by the current crisis. It touches upon a much more fundamental ‘ontological insecurity’ (Mitzen, 2006) or a loss of feeling or sense of oneself as a whole. Hence, whilst survey questions about job insecurity may have their utility, they do not capture the existential depth of the current crisis.

Abductive research may also be helpful here. We are faced with a global situation where existing research fails to provide possible answers to how we want and need to design future workplaces. What we know is that the COVID-19 crisis affects people in unequal ways, for instance, as demarcated by racial inequalities in society (McClure, Vasudevan, Bailey, Patel, & Robinson, 2020). To postulate a workplace that contributes to fairness, dignity and social justice, academics could engage more explicitly in abductive research. They could find ways through which (new) theory can work jointly with grounded reality. This may empower academics to engage in research that actively focuses on the creation of workplaces and policies that aim towards such goals as greater dignity and social justice in society.

One practical way through which this can be achieved is using dialogical, applicable social psychology (Mayo & La France, 1980), complemented with action research. Both tend to be rather underdeveloped types of research in work psychology. Work psychologists tend to assume that knowledge is created through survey research. They appear to ignore that knowledge is also generated by elders, indigenous people and through fictional narratives (e.g. Heron & Reason, 2008).

Bottom-up dialogical research strategies will allow researchers in work psychology to engage with people in the workplace from the moment of designing one’s research. It is imperative in these times that the actual benefactors of our research – people in the workplace – are integrated in the ways we do our research from the moment we formulate our research questions. Bottom-up dialogues with our stakeholders about the types of questions that are relevant to investigate and the ways we conduct our research not only benefit just the quality and relevance of our research but also useful to empower individuals in the workplace to contribute to goals such as social justice in the workplace.

Such dialogues can be combined with action research. In this way, academics do not just ‘observe’ what is going on in the workplace, but also develop, test and evaluate interventions with the participants with the shared goal of creating positive change in a given context. Co-creating action-focused research with other stakeholders in this way could contribute towards building greater social and environmental justice and dignity and fairness in workplaces.

Nonetheless, we also observe the general dislike amongst editors and reviewers in work psychology of qualitative research (e.g. Symon & Cassell, 2006). Firmly entrenched as a gateway to have research accepted in top academic journals is the antecedent-moderator-outcome research paradigm. Journal practices and cultures will have to change to allow a broader methodological set to be normalised in the field. With such a broader set of methodological tools, researchers may obtain a richer perspective of the impact of the current crisis and the ways workplaces can be developed in the future. Qualitative methods will allow for such research, which brings the lived experiences of workers to the fore, potentially developing more grounded theoretisation.

Pluralism in research will also contribute to a greater focus on Popperian ideals of falsification rather than verification. One such approach could be engaging in more case-study research (Eisenhardt, 1989). Eisenhardt suggested that case studies could be investigated through an iterative approach including continual iteration backward and forward. Each step in this research process involves cooperation amongst multiple stakeholders. Case study research includes multiple data collection methods and cross-case analysis. This could include (re)definition of the research question after each step and gathering some further evidence if needed. This would also promote more Popperian thinking – focusing on falsifying existing theory rather than merely searching for verification.

## Unheard voices in times of COVID-19

Since the beginning of the COVID-19 pandemic, work psychologists have turned to a growing body of research on teleworking, home-working and work–life balance (e.g. Bick, Blandin, & Mertens, 2020). Governments throughout the world called workers and organisations to work from home. However, this has often been done without taking into account the impossibility for many people worldwide to actually do this. Such exclusion is also still present in work psychology debates when it focuses on working from home.

## Have’s and have-not’s voices

The unheard voices in work psychology are rife. The criticism has often been voiced against the WEIRD nature of psychology (Schulz, Bahrami-Rad, Beauchamp, & Henrich, 2018). But even within wealthy countries, working from home remains a luxury that is only accessible to those who have office jobs and solid Internet connections at home. This excludes many casual workers, self-employed workers and others (Spreitzer, Cameron, & Garrett, 2017). Their voices remain largely unheard in work psychology. Similarly, the voices of people worldwide who have been dramatically affected by the current crisis remain invisible in our field. Amongst the unheard voices are not only women, older workers but also younger workers, people of mixed race, people with disabilities and other minority groups.

## Women and minority voices

The coronavirus, like most disasters, has unearthed societal vulnerabilities and inequalities regarding access to resources, capabilities and opportunities (Boin, Stern, & Sundelius, 2016) and further systematically disadvantaged certain groups, particularly women and minorities (Neumayer & Plümper, 2007). For instance, in academia, there is already a gender gap (Meyers et al., 2020; Pérez, 2019). The coronavirus disease 2019 has not affected all scientists equally. Women in academia publish less and are cited less than their male counterparts and since the coronavirus, overall publications by women have decreased by 16%, with a drop of 23% as first author and 16% as last author in the medical field (Gabster, Van Daalen, Dhatt, & Barry, 2020). Furthermore, women scientists with young dependents are the most disproportionately affected (Meyers et al., 2020). This also calls explicitly for an intersectional approach towards the unheard voices in our field.

Outside of academia, women are often employed in the informal sector. The informal sector has become an important job-creating sector (Fourie, 2018), outside of public and private organisations. However, there is little documented in work psychology about the dynamics of work in that space. The informal sector has often provided work to unskilled populations. Perhaps, the pandemic could also be seen as offering an opportunity to hear and learn from the experiences of that sector.

We therefore call for more space, understanding and research, for voices to be heard from a wide range of populations worldwide who are still neglected in work psychology. Those who do not fit the prototypical subject of psychological research – the white, university-educated, Western, heterosexual man – may be under-represented in terms of psychological explanation (Pérez, 2019). For instance, gender remains an important dimension in research. The current crisis has only amplified its importance, with women typically disadvantaged more by the crisis because of the disproportionate transfer of typical domestic responsibilities to them, such as household work, caring and child responsibilities. Moreover, issues of domestic violence have become particularly important in times of COVID-19, especially for those (generally) women and children for whom ‘home’ is not a safe place (Bradbury-Jones & Isham, 2020). In such cases, the notion of ‘working from home’ has to be problematised from many angles. Hence, we emphasise the importance to attend to unheard voices. Sometimes to leave the house and work outside of one’s home, even when exposing oneself to the risk of the virus, remains a safer option for individuals and their children.

## The denotation of vulnerability

This leads to the question of ‘vulnerability’, which is a central term in the current crisis. It seems as if even the denotation of someone being vulnerable is not decided by oneself exclusively but may be determined top-down. When governments or employers influence whether or not someone is fit enough to work because of lack of governmental support and whether one needs to be present at work during the pandemic, they will also determine vulnerability. We have witnessed many employers being generous and caring towards their employees. However, as the crisis continues, employees may be put under pressure to be present at work, even when they consider themselves vulnerable (to the virus, or more generally because of poor health or well-being). This raises the question about who gets to determine who is vulnerable and whether individuals have enough agency to take care of themselves and to protect themselves if needed or to engage in exposing themselves to risks in order to survive and make money or to protect others. Work psychologists need to be vigilant of such issues.

## Teaching in times of COVID-19

Finally, all the points made previously have implications for theory and methodology and also the teaching of work psychology. We recognise the pressures that academic teaching staff members are experiencing because of the pandemic. Some scholars lost their jobs, had their salary cut, doubled the number of students per class and had their employment contracts made more flexible and precarious. We or they must help prepare future generations of academics and work psychologists to think critically about the meaning of work that has been disrupted by the COVID-19 pandemic.

Students globally have suffered from the COVID-19 crisis. Many students are in the formative phases of their lives, developing themselves intellectually, emotionally and socially. The current crisis has diminished such opportunities for many students across the world. As work psychologists, we can also connect to our students and make their study a meaningful experience that they will take on for the rest of their lives. For instance, directly discussing with students how they are affected and how this has changed their perspectives on work may be an important endeavour.

Moreover, the above discussions about workers also apply to many students. Amongst students, there are those privileged with Internet access, stable connections and possibilities to study online. However, there are also less fortunate students who do not have such access. It is the responsibility of teachers and work psychologists to care for all students, and especially those with fewer privileges. Universities and psychologists can offer students additional mentoring and tutoring possibilities to cope with the current crisis.

Societal fault lines that have been manifested are amplified in the COVID-19 crisis. This is also present amongst our students. Thus, it is of great importance that universities put additional energy into maintaining the well-being of students. Especially, the students who lack the proper space, facilities, Internet connections, emotional support, moved out to study, live alone or in a violent neighbourhood, take care of others (children or the elderly), have certain health conditions or disabilities, and so on, may need additional care in the current crisis.

## A future of work and organisational psychology response to the COVID-19 crisis

This article has been written by authors affiliated with the FOWOP Movement for a more sustainable future for the field (see also www.futureofwop.com and Bal et al., 2019). This piece has evolved from a series of collective discussions by this group taking place since the start of the pandemic, in which we have reflected on how the pandemic has affected us as individuals, work psychology researchers and practitioners and members of different communities around the world. The FOWOP movement of academics in the work psychology field (and beyond) is formed out of frustration with contemporary practices and the lack of relevance and critical thought in the field. It aims to create better working conditions in academia, promote more relevant and critical research and address inequalities in the workplace and in academia. It published a manifesto in 2019 in the *European Journal of Work and Organizational Psychology*, which describes 10 responsibilities for academics in the field (Bal et al., 2019). The first, and perhaps the most fundamental responsibility, concerns the duty towards individuals in the workplace. Currently, the dominant practice in work psychology still remains embedded within the instrumental logic, favouring organisational profitability and survival over the well-being and dignity of individuals. The view of FOWOP is that individual well-being and dignity cannot be made instrumental towards organisational outcomes and profit. In the current crisis, this issue remains a fundamental issue in our practices as work psychologists.

In these days, global society debates the relationship between public health and economic survival, positioning a mutual exclusivity of protecting the health of (vulnerable) individuals versus protecting the economy. In this way, the health of individuals who might not suffer from the virus themselves, but from the effects of the lockdown, is overlooked. However, this debate can be perceived as an impossible paradox, whereby a genuine concern of governments for public health can be critiqued as an attack on the economy when a country is forced into a lockdown.

However, it is vital to understand the contemporary predicament as an aspect of globalised capitalism itself: the rapid spread of the virus across the world has been made possible because of the globalised economic system. Therefore, a narrow focus on the resurgence of the economic system itself, without postulating the necessity of changing the economic parameters (such as globalised supply chains, profitability and economic growth as ultimate priorities of an economy), is insufficient. Therefore, it is our responsibility to contribute to understanding and practical knowledge about more sustainable economic models and practices.

Hence, the question becomes how to change the economic system from within, from the level of the workplace, whereby balancing the needs for protection of vulnerable individuals, ensuring enough income or a basic income for citizens, protection of the environment and restoration of the planet and the transformation towards an entirely circular economy are all central. For work psychologists, this means to focus on the responsibilities we have as academics and practitioners. Drawing on these responsibilities as outlined in the manifesto (Bal et al., 2019), we suggest in this article dialogical routes, both theoretical and methodological, towards better understanding the workplace in the context of the COVID-19 crisis and towards dealing with the inequalities and suffering it brings upon workers.

We can translate such societal issues to the workplace by engaging in more research using non-traditional ontologies and methodologies, with systems approaches to understand behavioural responses to the crisis, thus empowering the unheard voice and those who are disproportionately affected, namely, women and minorities. Inductive and abductive research will also help to elucidate the dynamics underpinning contemporary workplaces and issues, and to articulate ways through which workplaces can become more inclusive and contribute to social and environmental justice, wealth distribution and dignity.

To do so, we as work psychologists also need to act more collectively, as issues in the workplace, both within and beyond academia, are too structural and interconnected to be solved by individuals or through individual research projects. For instance, precarious workers in academia need permanent staff to support them in their striving for their rights to remain employed during crises such as the current one. Affirmative action needs to be applied to give chances and correct bias. Moreover, colleagues of mixed race need white colleagues to address racism in the workplace, just as women colleagues need their men colleagues to do the same on their behalf. Thus, these issues and solutions are interconnected and dependent on collective action. Such collective initiatives also provide opportunities for mutual support and ways to connect with each other so that we can formulate actions together. In this way, we call for a ‘relational’ turn in work psychology, whereby the relationship between people becomes central in how we approach our research, our work and how we collaborate within academia. Collaboration instead of competition provides a healthier and more sustainable way forward to contribute to a fairer society and workplace.
